# Liver abscess complicated by sepsis due to *Yersinia enterocolitica*: a case report

**DOI:** 10.3389/fmed.2026.1822814

**Published:** 2026-09-30

**Authors:** Huanhuan Lin, Yuxiang Liu, Peng Ding, Lijia Zhi, Kunlan Long, Min Duan

**Affiliations:** 1 Chengdu University of Traditional Chinese Medicine, Chengdu, China; 2 Department of Critical Care Medicine, Hospital of Chengdu University of Traditional Chinese Medicine, Chengdu, China

**Keywords:** case report, liver abscess, meropenem, sepsis, *Yersinia enterocolitica*

## Abstract

*Yersinia enterocolitica* infection-induced liver abscess is extremely rare in clinical practice. It is usually only seen in specific high-risk groups, such as those with iron overload (such as hemochromatosis patients), immunosuppression (such as those undergoing chemotherapy, organ transplantation, or HIV infection), and those with poorly controlled diabetes. This case report presents a rare case of liver abscess complicated with sepsis caused by *Yersinia enterocolitica*. The pathogen was precisely identified through targeted next-generation sequencing of liver abscess fluid, combined with blood culture and abscess fluid culture, all of which consistently confirmed *Yersinia enterocolitica*. The treatment involved a comprehensive approach of percutaneous liver puncture and drainage combined with sensitive antibiotics, supplemented by dynamic imaging follow-up to monitor lesion changes. Eventually, the patient's condition significantly improved and they recovered.

## 1 Introduction

*Yersinia enterocolitica* is a foodborne zoonotic pathogen transmitted via the fecal–oral route. It can also be acquired through blood products, such as transfusion (1). Clinical manifestations are variable. Mild cases present as acute gastroenteritis. Some patients develop fever, right lower quadrant pain, and leukocytosis, mimicking acute appendicitis (pseudoappendicitis syndrome). Others may show extraintestinal manifestations, including reactive arthritis and erythema nodosum. Severe cases can progress to sepsis, with a mortality rate of up to approximately 50% (2). The disease lacks specific clinical features. Diagnosis mainly relies on pathogen culture and molecular testing of stool, blood, or pus.

Liver abscess caused by *Y. enterocolitica* is extremely rare. It mainly occurs in high-risk patients, such as those with iron overload (e.g., hemochromatosis), immunosuppression (e.g., chemotherapy, organ transplantation, HIV infection), or poorly controlled diabetes (3). In these patients, impaired host defenses or abnormal iron accumulation favor bacterial growth. Treatment usually combines percutaneous liver drainage with susceptible antibiotics, followed by serial imaging (4).

We report a case of *Y. enterocolitica* liver abscess complicated by sepsis. Long-term poor glycemic control was the key risk factor for this invasive infection. Targeted next-generation sequencing, blood culture, and pus culture all yielded *Y. enterocolitica*. The patient recovered markedly after percutaneous drainage, susceptible antibiotic therapy, and serial imaging monitoring. This case may deepen our understanding of *Y. enterocolitica* liver abscess and inform future treatment strategies.

## 2 Case presentation

A 56-year-old Tibetan man from a high-altitude region was admitted to a local hospital on October 28, 2025, for recurrent cough (Figure 1, timeline). He also had intermittent nausea and vomiting, constipation, and reduced urine output, without chills or high fever. Chest and abdominal CT showed scattered inflammation in both lungs and patchy slightly hypodense lesions in the liver. He was diagnosed with community-acquired pneumonia and treated with cefuroxime. During hospitalization, he developed high fever with chills, dyspnea, and palpitations. Ibuprofen induced profuse sweating without symptom relief, suggesting worsening infection. He was discharged and transferred to the emergency department of the Affiliated Hospital of Chengdu University of Traditional Chinese Medicine on November 4. He had type 2 diabetes for over 10 years, treated with oral metformin and glibenclamide without regular glucose monitoring; the latest HbA1c was 12.1% (November 4). He denied other chronic diseases, family genetic disorders, and drug or food allergies. He had a history of smoking, alcohol use, and unclean diet.

**Figure 1:**
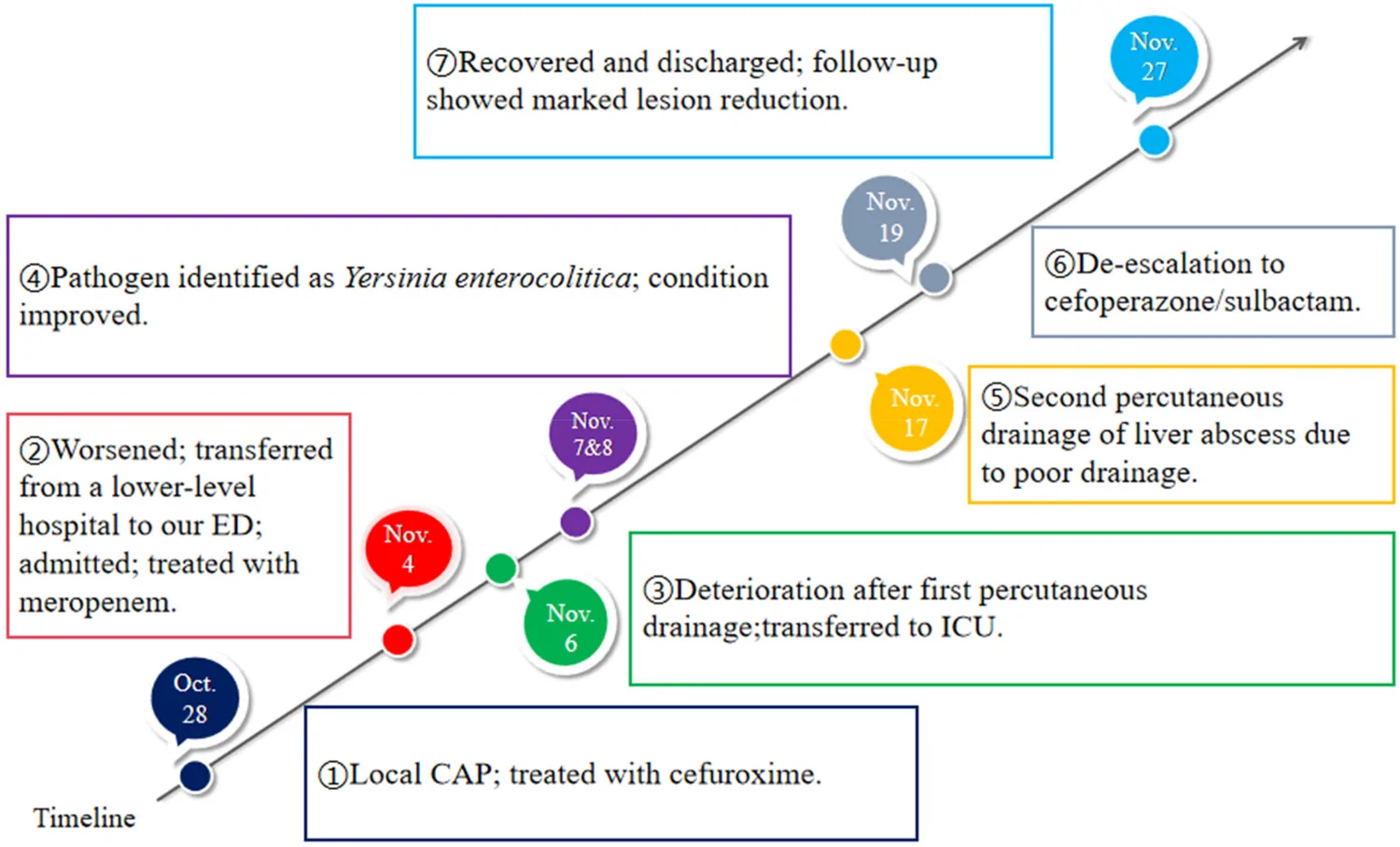
Timeline of the clinical course from admission to discharge and follow-up.

On November 4, he was admitted to the emergency department, alert but with chills, profuse sweating, and tachypnea. He also had oliguria and severe constipation (no bowel movement for 5 days, preceded by a transient episode of diarrhea 5 days earlier). Vital signs were unstable: temperature 36.3 °C, heart rate 120 beats/min, respiratory rate 20 breaths/min, and blood pressure 73/59 mmHg. Lung auscultation revealed coarse breath sounds and rales bilaterally. The abdomen was soft, without tenderness, muscle guarding, or rebound tenderness, and bowel sounds were present; however, percussion over the liver elicited tenderness. Laboratory findings are shown in Table 1. Unenhanced and contrast-enhanced abdominal CT revealed multiple low-density masses in the right hepatic lobe (multiple slightly hypodense lesions; the largest, in the right lobe, measured approximately 9.6 cm × 8.1 cm in cross-section with ill-defined margins) (Figures 2A,B). A preliminary diagnosis of liver abscess was made. Initial treatment included aggressive fluid resuscitation and meropenem. Source control was essential. On November 6, percutaneous drainage of the liver abscess was performed under ultrasound and digital subtraction angiography (DSA) guidance (Figure 2D, first lesion). About 180 mL of reddish-brown purulent fluid was aspirated (Figure 2C); contrast injection confirmed an irregular cavity, and a drainage catheter was placed. The pus sample was sent immediately for targeted next-generation sequencing (tNGS) to identify the pathogen. That evening, however, the patient deteriorated acutely, with sudden dyspnea and oxygen saturation dropping to 64%, accompanied by agitation, high fever, and chills. Heart rate rose to 160–190 beats/min, blood pressure to 189/105 mmHg, and respiratory rate to 33–35 breaths/min, with reduced urine output (300 mL/24 h) and right flank-abdominal pain. Given an identified source of infection and a SOFA score > 2, sepsis or impending septic shock was diagnosed, and the patient was urgently transferred to the intensive care unit (ICU).

**Table 1:** Laboratory test results of the patient.

Variable	Upon admission to the ICU	Transfer out of the ICU	Reference range
Blood Count and Inflammatory Markers			
White Blood Cell Count (10^9^/L)	12.45	7.77	3.5–9.5
Neutrophil Count (10^9^/L)	11.67	6.66	1.8–6.3
C-Reactive Protein (mg/L)	239.33	108.73	0–5
Procalcitonin (ng/mL)	36.22	2.29	0-0.05
Interleukin-6 (pg/mL)	3,125.49		0-4.4
Blood Biochemical Indicators			
Alanine Aminotransferase (U/L)	101	48	7–40
Aspartate Aminotransferase (U/L)	79	50	13–35
Creatinine (*μ*mol/L)	119.1	44.2	41–81
Urea (mmol/L)	6.45	4.72	3.1–8.8
Lactate Dehydrogenase (U/L)	213	193	120–250
High-Sensitivity Cardiac Troponin I (ng/mL)	0.01	＜0.002	0-0.0341
Myoglobin (ng/mL)	191.7	26	3-36
N-terminal Pro-B-type Natriuretic Peptide (pg/mL）	2,225.9	721.4	0-450
pH level	7.53	7.49	7.35–7.45
partial pressure of carbon dioxide（mmHg）	20.6	25.9	32–48
partial pressure of oxygen（mmHg）	125.3	63.9	83–108
Hematocrit（%）	37.9	35	36–53
lactic acid（mmol/L）	2.0	1.1	1.0–1.8
Bicarbonate（mmol/L）	17.4	19.9	21.4–27.3
base excess	-5.3	−3.5	−3–3
oxygenation index	208.33	96.5	
Coagulation function			
Prothrombin time（sec）	18.1	14.1	9.2–13.9
International Normalized Ratio	1.6	1.2	0.8–1.5
Activated Partial Thromboplastin Time（sec）	54.3	42.3	21.2–43.8
Fibrinogen（g/L）	5.25	6.01	2.0–4.0
D-dimer（ug/mL FEU）	12.72		0−0.55
Fibrinogen Degradation Products(ug/mL)	36.37		＜5

**Figure 2:**
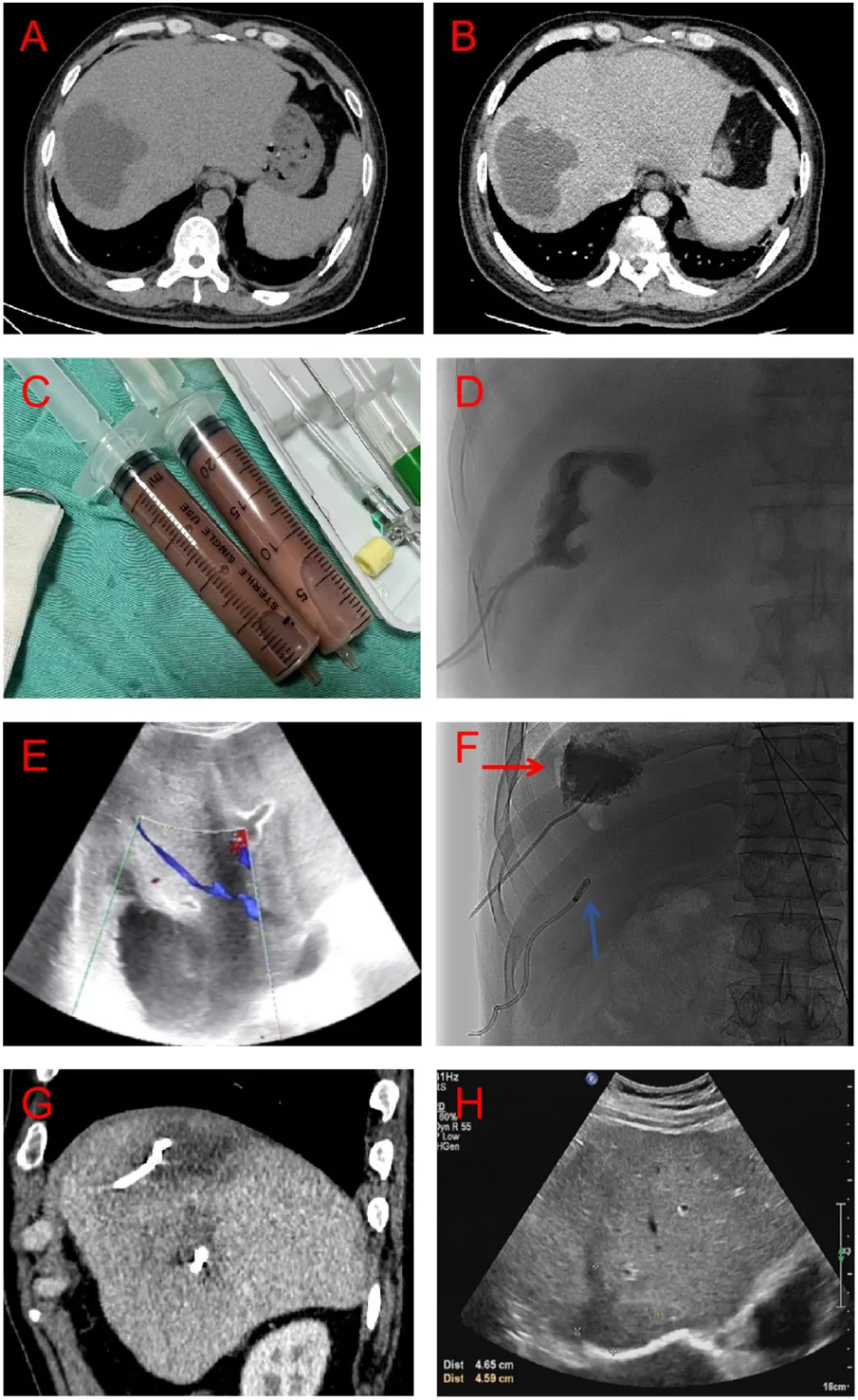
**(A,B)** abdominal CT (November 5). Normal liver size and morphology. A hypodense lesion in the right lobe measures approximately 9.6 × 8.1 cm, with ill-defined margins and mild peripheral enhancement after contrast. Hepatic abscess is suspected. **(C)** Percutaneous liver drainage fluid (November 6): reddish-brown, turbid, purulent exudate. **(D)** Percutaneous liver drainage (November 6): opacification of the abscess cavity during the procedure. **(E)** Ultrasound (November 16). A cystic-solid mixed-echoic mass in the right lobe measures 9.7 × 9.5 × 7.0 cm, with ill-defined borders and irregular shape. The center is anechoic, with scattered punctate echoes at the periphery. No significant blood flow is detected within the lesion. **(F)** Red arrow: abscess cavity from the second percutaneous transhepatic drainage (November 17). Blue arrow: abscess cavity from the first drainage (November 6). **(G)** Follow-up abdominal CT (November 21). Normal liver size and morphology. Multiple mass-like and patchy hypodense lesions in the right lobe, with heterogeneous density. The largest measures approximately 6.5 × 5.4 cm, with irregular shape, ill-defined margins, and mild peripheral enhancement after contrast. A drainage catheter is visible within the lesion. Compared with the November 5 CT, the lesions are markedly reduced in size. **(H)** Liver ultrasound at post-discharge follow-up (January 23, 2026). Scattered hypoechoic areas in the right lobe; the largest measures approximately 4.65*4.69 cm, with ill-defined borders and irregular shape, showing marked reduction compared with previous imaging.

After ICU admission on November 6, the patient received noninvasive ventilation. Blood, sputum, and abscess aspirate were collected for pathogen culture. Meropenem was continued, along with ongoing fluid resuscitation and vasoactive agents to maintain hemodynamic stability. The pus tNGS report (November 6) detected only human herpesvirus 4 (1,897 sequences; estimated concentration 10^3^; low confidence), which could not explain the clinical deterioration. On November 7 and 8, blood and liver abscess aspirate cultures both grew *Y. enterocolitica* (susceptibility results in Supplementary Material). We then consulted the sequencing company and selected ultra-multiplex PCR amplicon sequencing with a broader detection range for the pus sample. It also detected *Y. enterocolitica* (concentration 4 × 10⁶ copies/mL; signal strength high; 223,689 sequences), confirming the etiological diagnosis (detailed tNGS results in Supplementary Material). After targeted treatment, the patient improved rapidly and vital signs stabilized. He was transferred from the ICU to the general ward on November 10.

In the general ward, meropenem was continued. Inflammatory markers and liver function gradually normalized. The drainage fluid changed from reddish-brown purulent to clear yellow, and its volume gradually decreased. However, follow-up ultrasound on November 16 showed no significant reduction in the abscess (Figure 2E), suggesting a multiloculated abscess or incomplete drainage. On November 17, a second ultrasound- and DSA-guided percutaneous drainage was performed (Figure 2F, second lesion), yielding about 120 mL of reddish-brown turbid fluid. After the second drainage, follow-up ultrasound confirmed abscess shrinkage. The treatment strategy was then adjusted to continuous catheter drainage with saline irrigation of the cavity. Given the stable condition and confirmed pathogen, antibiotics were de-escalated to cefoperazone-sulbactam on November 19. Contrast-enhanced abdominal CT on November 21 (Figure 2G) showed marked lesion shrinkage. The drainage catheter was removed uneventfully on November 22. The patient was discharged fully recovered on November 27.

## 3 Follow-up

On January 23, 2026, follow-up liver ultrasound showed a residual lesion measuring 4.65 × 4.69 cm (Figure 1H). Other blood tests were unremarkable.

## 4 Discussion

### 4.1 General epidemiology and infectious routes of pyogenic liver abscess

Pyogenic liver abscess (PLA) accounts for approximately 80% of all liver abscesses (5). Its pathogen spectrum varies markedly by region. In Europe and North America, *Escherichia coli* and *Streptococcus* are the most common pathogens. In the Asia-Pacific region, *Klebsiella pneumoniae* has become the leading cause. In Taiwan, China, the spread of hypervirulent *K. pneumoniae* clones has raised the PLA incidence to 17.6 per 100,000 persons (6). In recent years, with increased migration and improved diagnostics, *K. pneumoniae*-associated PLA has also been increasingly reported in Europe and North America (7).

Independent risk factors for PLA include diabetes, cirrhosis, biliary tract disease, and a history of gastrointestinal surgery (8). Among these, diabetes impairs host immune defenses and plays a key role in abscess risk (9, 10).

Pyogenic liver abscess can be classified into five categories based on the origin of infection; this classification guides microbiological assessment and subsequent treatment (11): (i) Biliary route — the most important cause of bacterial liver abscess. It results from bile stasis secondary to benign or malignant biliary obstruction (e.g., gallstones, congenital anomalies), which promotes microbial proliferation and invasion of the liver parenchyma. (ii) Portal venous route — bacteria enter the liver via the portal vein, causing pylephlebitis or infectious suppurative thrombosis, secondary to any intra-abdominal infectious process (e.g., bowel content leakage, peritonitis). (iii) Bacteremic route — bacteria invade the liver via the hepatic artery or portal vein from a distant focus (usually endocarditis, pneumonia, or osteomyelitis). If *Streptococcus* or *Staphylococcus* is isolated, an extra-abdominal source with hematogenous spread to the liver should be considered. (iv) Direct extension route — infection spreads from adjacent organs to the liver. (v) Traumatic route — caused by penetrating injury or complications of blunt trauma. Different infectious routes often correspond to different pathogen spectra and anatomical distributions. For example, portal-vein-derived abscesses are commonly located in the right hepatic lobe, related to the anatomical dominance of the right portal vein branch (12).

### 4.2 Specific clinical and molecular features of *Yersinia enterocolitica* liver abscess

Compared with *K. pneumoniae* or *E. coli*, liver abscess caused by *Y. enterocolitica* is extremely rare. Globally, the literature consists mainly of case reports and small-sample reviews (13). However, this pathogen has several unique clinical and molecular features that enable it to breach the intestinal barrier and establish infection in the liver in specific hosts.

Regarding the infectious route, *Y. enterocolitica* liver abscess almost always occurs via the portal venous route. The bacterium mainly invades the terminal ileum and colon. After crossing the intestinal epithelium through M cells, it colonizes and multiplies in Peyer's patches and mesenteric lymph nodes. It is then transported by macrophages or dendritic cells to the mesenteric venous system and disseminated to the liver via the portal vein (14, 15).

Regarding host risk factors, the most prominent susceptibility factor for *Y. enterocolitica* liver abscess is abnormal iron metabolism. Iron overload states caused by hereditary hemochromatosis, repeated blood transfusions, chronic kidney disease, and deferoxamine therapy can significantly enhance *Y. enterocolitica* virulence and the risk of systemic infection (16). The molecular mechanism is as follows (17): iron is a limiting nutrient for bacterial growth; exogenous siderophores (e.g., deferoxamine) or host iron overload can promote systemic dissemination of *Yersinia*. Besides iron overload, advanced age, immunodeficiency, diabetes, and malignancy are also known risk factors for systemic *Y. enterocolitica* infection (18).

Regarding molecular pathogenesis (19), the core virulence arsenal of pathogenic *Y. enterocolitica* includes the approximately 70-kb *pYV* virulence plasmid and multiple chromosomal virulence genes. The *pYV* plasmid encodes the type III secretion system (T3SS) and its effector proteins Yops (YopH, YopE, YopT, YopP, YopM, etc.), which inhibit phagocytosis by phagocytes, block pro-inflammatory signaling pathways, and induce macrophage apoptosis. We speculate that this is the key mechanism enabling the bacteria to resist killing by Kupffer cells and neutrophils in the hepatic sinusoids. The chromosomally encoded invasin (*InvA*) and attachment-invasion locus protein (*Ail*) mediate bacterial adhesion to and internalization by the intestinal epithelium. We estimate that they may also participate in adhesion to hepatic sinusoidal endothelium and hepatocytes. The *pYV*-encoded *YadA* adhesin mediates both serum resistance and the tight bacterium–host contact required for T3SS injection.

In summary, the ability of *Y. enterocolitica* to reach the liver via the portal vein after intestinal invasion and establish infection depends on the virulence genes at each of the above stages. Although some stages have not been verified, we reasonably hypothesize the following (Figure 3): after intestinal invasion, chromosomally encoded *InvA* and *Ail* bind to *β*1 integrins on the surface of intestinal epithelial cells and M cells, mediating bacterial adhesion and internalization and enabling breach of the intestinal mucosal barrier. During dissemination to the liver via the portal vein, *pYV*-encoded *YadA* and *Ail* jointly mediate serum resistance (inhibiting complement-mediated killing), ensuring bacterial survival in the bloodstream and portal venous system without clearance. After reaching the hepatic sinusoids, *InvA*, *Ail*, and *YadA* further mediate bacterial adhesion to and internalization by hepatic sinusoidal endothelial cells and hepatocytes, enabling colonization in the liver as the target organ. However, adhesion and internalization alone do not guarantee survival. The bacteria immediately face phagocytic killing by Kupffer cells and neutrophils in the hepatic sinusoids. At this point, the *pYV*-encoded T3SS becomes a key immune evasion tool: by injecting effector proteins Yops (YopH, YopE, YopT, YopP, YopM, etc.), it inhibits phagocytosis, blocks pro-inflammatory signaling, and induces macrophage apoptosis, thereby enabling the bacteria to survive, multiply, and ultimately form abscesses in the hepatic sinusoids and parenchyma.

**Figure 3:**
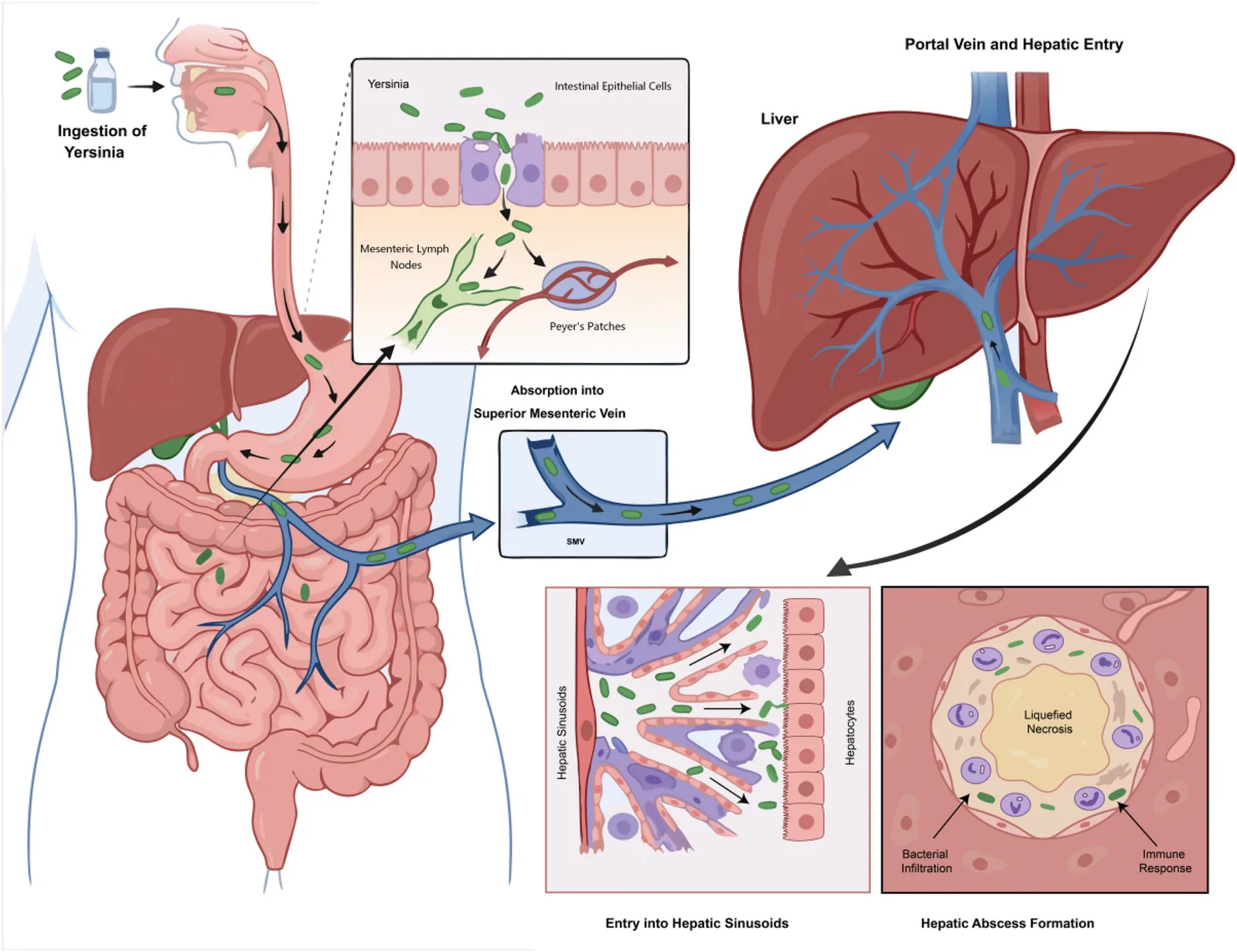
Schematic illustration of *Yersinia enterocolitica* dissemination, hepatic sinusoid invasion, and liver abscess formation. SMV, superior mesenteric vein.

*Y.enterocolitica* can be divided into six biotypes (1A, 1B, 2, 3, 4, and 5) and more than 60 serotypes (2). The high-pathogenicity island (HPI) distinguishes highly pathogenic from low-pathogenic strains (17). At the biotype level, biotype 1B is the most virulent and most directly associated with liver abscess because it carries both *pYV* and HPI. Biotypes 2–5 lack HPI but can still cause similar extraintestinal infections in iron-overloaded or immunocompromised hosts (13).

### 4.3 Inference of the infectious route and analysis of risk factors in this patient

First, the infectious route is highly consistent with portal venous dissemination. The abscess was located in the right hepatic lobe, matching the typical anatomical distribution of portal-vein-derived liver abscess. The pathogen was *Y. enterocolitica*, an intestinal pathogen that mainly invades the terminal ileum and colon. The patient reported transient diarrhea, suggesting a preceding intestinal mucosal infection. Based on these three points, we hypothesize the following infectious process: the bacteria were first ingested orally and caused intestinal mucosal infection, then penetrated the intestinal wall into the mesenteric venous system, and disseminated via the right portal vein branch to the right hepatic lobe to form an abscess. This inference is consistently supported by both imaging localization and the biological characteristics of the pathogen.

Second, the patient had multiple known risk factors. He was an ethnic minority from a high-altitude, cold region. *Y. enterocolitica* can survive in low-temperature environments and spread through contaminated meat and dairy products (1). However, the patient could not recall the specific foods consumed before onset; based on his living environment and local cultural dietary habits, we can only speculate that he consumed contaminated meat, dairy products, or other foods. More importantly, the patient had type 2 diabetes with poor glycemic control. Hyperglycemia can impair neutrophil function and is a shared risk factor for both PLA and systemic *Y. enterocolitica* infection (10). In addition, the patient's serum ferritin before discharge was > 2000ng/mL (reference range 30–400 ng/mL), markedly elevated, suggesting possible abnormal iron metabolism. Although it is impossible to fully distinguish whether this elevation resulted from underlying iron overload, hepatocyte necrosis during liver abscess, or a combination of both, abnormal iron metabolism itself is a hallmark susceptibility factor for *Y. enterocolitica* liver abscess (16). The patient had no history of repeated blood transfusions, chronic liver disease, or hereditary iron metabolism disorders. However, his type 2 diabetes may be associated with long-term abnormal iron metabolism. Studies have shown that elevated serum ferritin is positively correlated with the risk of type 2 diabetes; in Chinese adults, ferritin > 140 ng/mL is associated with a significantly increased diabetes risk (20). This patient's ferritin far exceeded this threshold, suggesting possible long-term abnormal iron metabolism, and type 2 diabetes may be one of its metabolic manifestations.

Third, the rarity of this case is noteworthy. Currently, case reports of *Y. enterocolitica* liver abscess are extremely rare globally. Prevalence and molecular epidemiological data in China are insufficient, and studies on virulence factors of *Y. enterocolitica* isolated from liver abscesses are even scarcer. This case report provides valuable reference for clinical understanding of the infectious route, risk factors, and imaging features of this disease.

### 4.4 Imaging diagnosis

The clinical and biological symptoms of liver abscess are nonspecific. Diagnosis relies on pathogen testing and imaging. Ultrasound has a sensitivity of 86%–96%, and CT has a sensitivity > 90%; both are the main tools for confirming liver abscess (11). Other important features observable on imaging include abscess location, size, number, consistency, and the presence of gas within the abscess. These radiological features help classify liver abscess and select the most appropriate treatment. Advances in imaging technology have led to more efficient diagnosis and reduced mortality.

### 4.5 Treatment strategy and considerations

For bacterial liver abscess, the main treatment is systemic antibiotic therapy combined with percutaneous drainage. First, for liver abscess with septic shock/MODS: the initial empiric antimicrobial regimen can be a carbapenem (e.g., imipenem-cilastatin, meropenem, biapenem, etc.). The antibiotic course is 7–10 days; after clinical improvement or infection resolution, de-escalation therapy is performed. Regarding drug resistance, studies indicate that *Y. enterocolitica* typically possesses intrinsic *β*-lactamase (21). Some studies show increasing resistance to clinically relevant antibiotics, including trimethoprim-sulfamethoxazole and tetracycline (22). Savin et al. reported changes in quinolone resistance in this bacterium (23). Therefore, we should be alert to drug resistance and use antibiotics rationally.

Second, ultrasound- or CT-guided percutaneous catheter drainage of liver abscess is an important treatment method for bacterial liver abscess and should be performed as early as possible. The advantages of percutaneous catheter drainage include convenience, safety, effectiveness, minimal invasiveness, and high patient acceptance. Even for difficult-to-treat multiple bacterial liver abscesses, the treatment success rate exceeds 90%. Giant abscesses (diameter > 10 cm) can also be treated by catheter drainage; for patients unable to tolerate surgery, multiple drainage catheters can be placed to achieve satisfactory results, but such patients have a higher risk of treatment failure and other complications (24). For surgery, even when surgical indications exist (e.g., large abscess volume, anatomically inaccessible location, multiple comorbidities, or other complex factors), the risk–benefit ratio should be carefully considered. Many studies have found no significant difference in mortality between percutaneous drainage (PD) and stent drainage (SD) (10). In addition, if drainage of a malignant abscess is ineffective, percutaneous drainage can still be performed if the abscess is accessible, given its minimally invasive nature. It is worth noting that infection with this bacterium is an infectious disease that poses a public health threat. Prevention may be an important approach; studies have even constructed vaccines against *Yersinia*, which, if effective, would be of great significance for reducing the incidence of complications such as liver abscess (25, 26).

In summary, after the patient's first percutaneous drainage, sepsis worsened and caused acute respiratory distress syndrome. Studies have found that in patients with bacterial liver abscess > 10 cm in diameter, 25% develop complications after percutaneous drainage, including sepsis, pleural effusion, and the need for repeat percutaneous drainage (24). However, after bundle therapy, the patient's sepsis was rapidly corrected. After reassessment, a second percutaneous drainage was performed with good drainage and no complications.

### 4.6 Prognosis and follow-up

Regular follow-up is performed, with the duration determined by abscess size. Clinical follow-up indicators include symptoms, body temperature, white blood cell count, and serum inflammatory markers such as CRP and PCT. Imaging follow-up is performed only when clinical symptoms persist or drainage is inadequate; imaging resolution is much slower than improvement in clinical symptoms and biochemical markers. One study followed 102 patients with bacterial liver abscess and found that the mean ultrasound resolution time was 16 weeks for abscesses < 10 cm and 22 weeks for abscesses > 10 cm (27). We followed the patient monthly. Outpatient liver ultrasound showed progressive abscess shrinkage, and all laboratory indicators were normal.

### 4.7 Limitations

First, as a single case report, a direct relationship between *Y. enterocolitica* and liver abscess cannot be established; more cohort studies are needed for verification. Second, the diagnosis of *Y. enterocolitica* liver abscess currently relies solely on pathogen results, lacking clear imaging or laboratory diagnostic criteria. Third, systematic comparison of literature benchmark data with observed recovery kinetics would help strengthen the assessment of long-term prognosis. However, as a single case report, limited by the small sample size and the lack of standardized longitudinal imaging follow-up at fixed intervals, a formal quantitative comparison could not be performed. Finally, limited by the scope of the genetic database, bioserotype information could not be obtained, precluding further exploration of molecular mechanisms.

## 5 Conclusion

This report may prompt clinicians to consider *Y. enterocolitica* as a potential, albeit rare, etiological agent of pyogenic liver abscesses in patients with uncontrolled diabetes or iron overload. Meanwhile, more mechanistic studies on *Y. enterocolitica* liver abscess are needed to improve understanding and provide precise, effective treatment strategies for patients.

## Data Availability

The original contributions presented in the study are included in the article/Supplementary Material, further inquiries can be directed to the corresponding author.
