# Supplementary material for: Liver abscess complicated by sepsis due to Yersinia enterocolitica: a case report
Source: Front Med (Lausanne). 2026 Sep 30;13:1822814. doi: 10.3389/fmed.2026.1822814 (PMC13661410; doi:10.3389/fmed.2026.1822814)
Supplement: Supplementary file 1 [file Table1.docx]

Table 1. Targeted Next-Generation Sequencing Report Results

| Item | Name | Concentration in Sample | Signal Intensity (Sequence Count) |
| --- | --- | --- | --- |
| Bacteria Detected | *Yersinia_enterocolitica* | 4x10^6^ copies/ml | High (223,689) |
| Fungi Detected | None | - | - |
| Viruses Detected | None | - | - |
| Other Pathogens | None | - | - |
| Suspected Background Microorganisms | None | - | - |
| Gray-Zone Signal List | None | - | - |
| Resistance and Virulence Genes | None | - | - |

Table 2. Targeted Next-Generation Sequencing Quality Control Results

| QC Parameter | | QC Result |
| --- | --- | --- |
| Experimental QC | Library Concentration | Pass |
|  | Contamination Prevention Label | Pass |
|  | Internal Reference | Pass |
|  | Negative Control | Pass |
| Data QC | Q30(%) | 96.94 |
|  | Total Sequence Count | Pass |

Table 3. Targeted Next-Generation Sequencing Data

| name | 1 | 2 | 3 | 4 | 5 |
| --- | --- | --- | --- | --- | --- |
| Sample ID | - | - | - | - | - |
| Total reads | - | - | - | - | - |
| High quality reads (Q30) | - | - | - | - | - |
| Dimmer | - | - | - | - | - |
| Clean reads | - | - |  |  |  |
| Total Align | - | - | - | - | - |
| Real Align | - | - | - | - | - |
| DNA Concentration | - | - | - | - | - |
| Sample Type | - | - | - | - | Pus |
| Library Concentration |  |  |  |  |  |
| *Yersinia enterocolitica*, N127_12_s1 gene="*amiB*" | 1 | 0 | 0 | 2 | 568146 |
| *Yersinia enterocolitica*, N135_01 gene="*mglA*" | 1 | 0 | 0 | 0 | 231354 |
| *Yersinia enterocolitica*, N135_01_s1 gene="*ygaH*" | 0 | 0 | 0 | 0 | 293932 |
| *Yersinia enterocolitica*, N15_044 gene="*leuA*" | 0 | 4 | 1 | 2 | 936612 |

Table 4. Routine Antimicrobial Susceptibility Testing of Pus

| Antibiotic | MIC(μg/ml) | KB(mm) | Susceptibility |
| --- | --- | --- | --- |
| Levofloxacin | 0.5 |  | S |
| Meropenem | ＜=0.25 |  | S |
| Minocycline | 2 |  | S |
| Ertapenem | ＜=0.12 |  | S |
| Cefoxitin | ＞=64 |  | R |
| Gentamicin | ＜=1 |  | S |
| Tigecycline | ＜=0.5 |  | S |
| Tobramycin | ＜=1 |  | S |
| Eravacycline | 0.25 |  | S |
| Ampicillin/Sulbactam | 8 |  | S |
| Cefoperazone/Sulbactam | ＜=8 |  | S |
| Trimethoprim-Sulfamethoxazole | ＜=20 |  | S |
| Cefazolin | 64 |  | R |
| Chloramphenicol | 16 |  | I |
| Ceftazidime | ＜=0.12 |  | S |
| Aztreonam | ＜=1 |  | S |
| Doxycycline | 2 |  | S |
| Amikacin | ＜=1 |  | S |
| Cefuroxime | 4 |  | S |
| Cefuroxime Axetil | 4 |  | S |
| Colistin | ＜=0.5 |  | S |
| Ciprofloxacin | 0.5 |  | I |
| Ceftriaxone | ＜=0.25 |  | S |

S: Susceptibility; I: Intermediate; R: Resistant; MIC: Minimal Inhibitory Concentration; KB: Kirby-Bauer.

Table 5. Routine Antimicrobial Susceptibility Testing of Blood

| Antibiotic | MIC(μg/ml) | KB(mm) | Susceptibility |
| --- | --- | --- | --- |
| Ertapenem | ＜=0.12 |  | S |
| Cefoxitin | ＞=64 |  | R |
| Doxycycline | 1 |  | S |
| Cefuroxime Axetil | 4 |  | S |
| Trimethoprim-Sulfamethoxazole | ＜=20 |  | S |
| Gentamicin | ＜=1 |  | S |
| Ampicillin/Sulbactam | 4 |  | S |
| Cefoperazone/Sulbactam | ＜=8 |  | S |
| Minocycline | 2 |  | S |
| Levofloxacin | 0.5 |  | S |
| Meropenem | ＜=0.25 |  | S |
| Cefuroxime | 4 |  | S |
| Tigecycline | ＜=0.5 |  | S |
| Amikacin | 2 |  | S |
| Tobramycin | ＜=1 |  | S |
| Cefazolin | 64 |  | R |
| Eravacycline | 0.25 |  | S |
| Aztreonam | ＜=1 |  | S |
| Ceftriaxone | ＜=0.25 |  | S |
| Colistin | ＜=0.5 |  | S |
| Ciprofloxacin | 0.5 |  | I |
| Chloramphenicol | 16 |  | I |
| Ceftazidime | ＜=0.12 |  | S |

S: Susceptibility; I: Intermediate; R: Resistant; MIC: Minimal Inhibitory Concentration; KB: Kirby-Bauer.
